# Efficacy of a far ultraviolet-C light technology for decontamination of surfaces and personal items in long-term care facility resident rooms

**DOI:** 10.1017/ash.2026.10345

**Published:** 2026-05-19

**Authors:** Amelia L. Milner, Samir Memic, Maria M. Torres-Teran, Jennifer L. Cadnum, Curtis Donskey

**Affiliations:** 1 VA Northeast Ohio Healthcare System, USA; 2 Infectious Diseases Section, Louis Stokes Cleveland VA Medical Center, Cleveland, USA

## Abstract

Contaminated surfaces in long-term care facility resident rooms may contribute to transmission of healthcare-associated pathogens. We demonstrated that a far ultraviolet-C light technology operated for 2 to 4 hours during periods when residents were out of the room was effective in reducing contamination of surfaces and personal items.

## Introduction

Long-term care facility (LTCF) residents have been linked to transmission of pathogens such as *Clostridioides difficile*, multidrug-resistant Gram-negative bacilli, and methicillin-resistant *Staphylococcus aureus* (MRSA).^1,2^ Colonized patients can directly transfer pathogens to personnel or to surfaces during activities outside their room,^3–5^ and organisms on high-touch surfaces and floors can be transferred outside the room.^6,7^ Daily disinfection of surfaces in LTCF rooms could potentially reduce the risk for transfer of pathogens outside the room. However, manual cleaning and disinfection of resident rooms during admission is challenging because patient care and personal items are often placed on surfaces and may not be amenable to use of liquid disinfectants.

Ultraviolet-C (UV-C) light could potentially be useful for decontamination of surfaces including patient care and personal items when LTCF residents are out of their rooms.^8^ However, room devices using 254-nm UV-C require a substantial amount of time to operate and are hazardous if inadvertent exposure occurs.^8^ Far UV-C light (200–230 nm) is an alternative technology that may be safe to operate continuously in occupied spaces.^8^ Far UV-C devices can also be operated only when rooms are not occupied though manual on-off switches or using motion sensors that discontinue exposure when movement is detected.^9^ Far UV-C light devices are typically ceiling- or wall-mounted so staff time to operate the devices is minimized. We previously demonstrated that a far UV-C technology reduced MRSA by ≥1.7 log_10_ at multiple sites in a patient room with a 45-minute exposure.^9^ Here, we conducted a pilot study to assess the efficacy of the far UV-C technology for decontamination of real-world surfaces and items in unoccupied LTCF rooms.

## Methods

### Description of the far UV-C light technology

The wall-mounted far UV-C technology (Mynatek, Inc., Oakland, CA) uses 3 krypton-chloride excimer lamps emitting a primary wavelength of 222 nm.^9^ The device includes sensors that detect people within the field of illumination. For the purposes of the study, the devices were programmed to automatically discontinue far UV-C light delivery whenever people were detected and to automatically resume delivery when they moved outside the area of exposure.

### Evaluation of the far UV-C technology for decontamination of surfaces and patient and personal care items in rooms of LTCF residents

**Figure 1.**
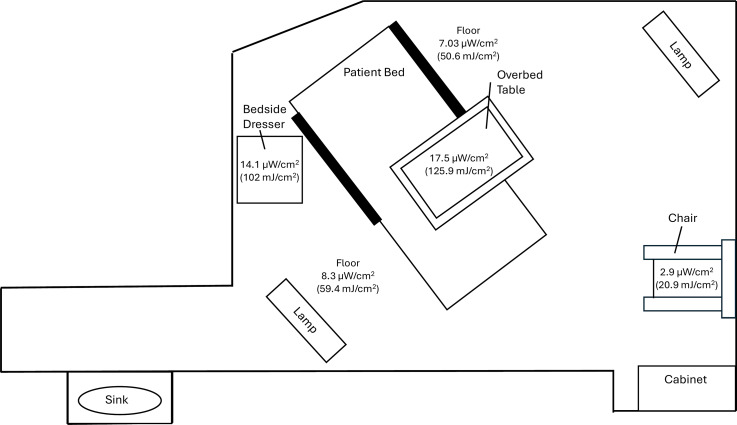
Diagram of a typical long-term care facility resident room including the positions of the far ultraviolet-C (UV-C) devices and irradiance readings plus cumulative doses of far UV-C delivered after 2 hours in 5 room locations.

The study was approved by the Cleveland VA Medical Center’s Institutional Review Board. We evaluated the efficacy of the far UV-C technology during periods when LTCF residents in single-occupancy 38 m^3^ rooms were out of the room. The devices were mounted on metal poles. Figure 1 provides a diagram of a typical LTCF room with the devices in position. Two devices were positioned at a height of 2 meters at opposite sides of the room with the bed between the devices. For a typical room, a radiometer (UIT2400 Handheld Light Meter for 222 nm; Ushio America, Cypress, CA) was used to measure irradiance in µW/cm^2^ at 5 locations. The far UV-C doses in mJ/cm^2^ were calculated for a 2-hour exposure time.

BBL culture swabs (Becton Dickinson, Sparks, MD) premoistened with Dey-Engley neutralizer were used to sample 10 room surfaces at baseline and after 2 hours; the surfaces were sampled after 4 hours when feasible. Surfaces included high-touch surfaces (eg bed rail, bedside table, call button, chair armrest, telephone), personal items (eg books, magazines, physical therapy equipment), and the floor adjacent to the bed. Separate swabs were used for each surface; average results were calculated for floors, high-touch surfaces, and personal items. The location of the sampling sites was not standardized as surfaces and items were in variable locations and were not re-positioned for the study. For larger surfaces, separate 10 × 10 cm adjacent areas were sampled at each time point; for smaller surfaces, one-third of the entire surface area was sampled at each time point. The swabs were processed for identification and enumeration of total heterotrophic colony counts and pathogens, including *S. aureus,* enterococci, and Gram-negative bacilli.^9^

## Data analysis

The primary outcome was the total CFU of pathogens (composite of *S. aureus*, enterococci, and Gram-negative bacilli) before versus after far UV-C exposure. To compare CFU recovered, a mixed-effects negative binomial regression model with adjustment for time and organism type was used. Analyses were performed in R version 4.2.2 (R Foundation for Statistical Computing, Vienna, Austria).

## Results

Figure 1 shows the far UV-C irradiance readings and UV-C doses calculated for a 2-hour exposure at 5 sites in a typical LTCF room. Irradiance readings varied from 2.9 µW/cm^2^ on the seat of a chair in the corner of the room not in direct line of site of either device to 17.4 µW/cm^2^ on an overbed table in direct line of site of both devices.

Ten ambulatory LTCF residents participated. At baseline, 37 of 100 (37%) sites sampled were contaminated with 1 or more pathogens, including 9 (9%) with *S. aureus*, 17 (17%) with enterococci, and 11 (11%) with Gram-negative bacilli. Three of 9 (33%) *S. aureus* isolates were MRSA and 3 of 17 (17%) of the enterococci were vancomycin-resistant *Enterococcus faecium*.

**Figure 2.**
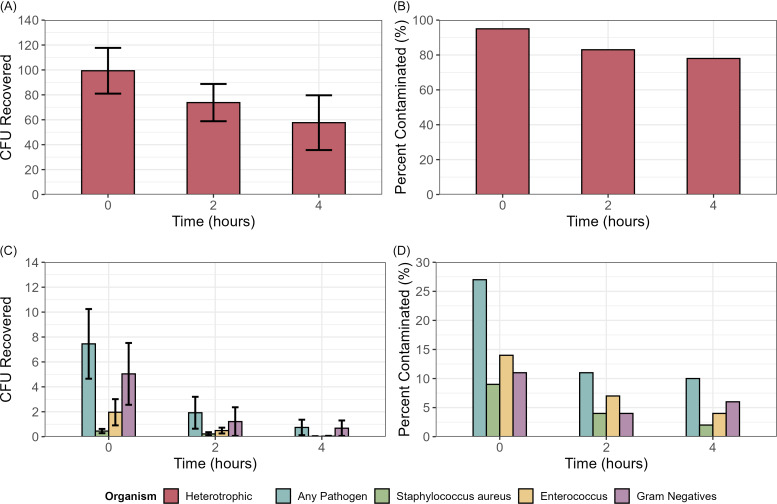
Effectiveness of the far ultraviolet-C (UV-C) light technology in reducing bacterial contamination on surfaces and patient and personal care items in long-term care facility resident rooms. (A) mean colonies of heterotrophic organisms recovered on nonselective blood agar plates, (B) percent positive recovery of heterotrophic organisms, (C) mean colonies of healthcare-associated pathogens recovered, and (D) percent positive recovery of healthcare-associated pathogens. CFU, colony-forming unit. Error bars show standard error.

As shown in Figure 2, far UV-C exposure reduced total pathogen counts and total heterotrophic counts on surfaces. Prior to far UV-C exposure, floors had higher levels of contamination (mean CFU, heterotrophs 417.3 and total pathogens 52.7) than personal items (mean CFU, heterotrophs 141.3, and total pathogens 1.5) or high-touch surfaces (mean CFU, heterotrophs 80.0, and total pathogens 2.3). For total pathogens including all sites, CFU counts at 2 hours were reduced with an estimated rate ratio (RR) of 0.29 (95% CI 0.14, 0.61; *P* = .001) indicating a 71% reduction relative to baseline; at 4 hours a similar degree of reduction was present (RR 0.26, 95% CI 0.1, 0.71, *P* = .005) corresponding to a 74% decrease from baseline. The percentage of sites with contamination with 1 or more pathogens was significantly reduced after 2 (27% vs 12%; *P* = .01) and 4 hours (27% vs 10%; *P* = .003).

## Discussion

Our results suggest that the far UV-C technology could potentially be a useful adjunctive measure for decontamination of LTCF resident rooms. A single 2-hour daily exposure may be sufficient to reduce contamination as this was as effective as a 4-hour exposure. Alternatively, the device could be operated continuously or intermittently throughout the day when residents are outside the room to address ongoing shedding of pathogens.

Far UV-C light has advantages and disadvantages in comparison to 254-nm UV-C. Far UV-C is safer than 254-nm UV-C and can be operated continuously in occupied spaces or only when people are not present.^9,10^ Far UV-C light technologies require longer exposure times. Far UV-C devices installed in the ceiling would require minimal time to operate, but installation in many rooms might be cost-prohibitive.

This study has some limitations. The study was conducted in 1 LTCF. We did not audit daily room cleaning during the study. However, previous fluorescent marker audits in our LTCF have demonstrated that daily cleaning is suboptimal (<25% of high-touch surfaces cleaned). We only assessed efficacy of the technology in reducing contamination. Future studies are needed to examine the feasibility of implementation in LTCFs and the impact on transfer of pathogens outside of rooms.

## Conclusion

An automated far UV-C technology reduced real-world contamination in LTCF rooms. Additional studies are needed to determine if adjunctive use of far UV-C light will reduce transfer of pathogens from patient rooms.
